# Data-Driven Tailoring Optimization of Thermoset Polymers Using Ultrasonics and Machine Learning

**DOI:** 10.3390/polym17070895

**Published:** 2025-03-27

**Authors:** Gonzalo Seisdedos, Milo G. Prisbrey, Pavel Vakhlamov, Joshua Fernandez, Riangello De Freitas, Tommy Rockward, Eric S. Davis

**Affiliations:** 1 Materials Physics and Applications (MPA-11), Los Alamos National Laboratory, Los Alamos, NM 87545, USA; mprisbrey@lanl.gov (M.G.P.); pvakhlamov@lanl.gov (P.V.); trock@lanl.gov (T.R.); esdavis@lanl.gov (E.S.D.); 2 Mechanical and Materials Engineering Department, Florida International University, Miami, FL 33174, USA; jfern604@fiu.edu (J.F.); rdefr007@fiu.edu (R.D.F.)

**Keywords:** thermosets, resins, ultrasonics, non-destructive evaluation, cure kinetics, elastic properties, machine learning

## Abstract

Thermoset polymers are highly demanded for their structural robustness, thermal stability, and chemical resistance. Tailoring the properties of these polymers for high-performance applications is often preferred to designing brand-new polymers. However, the traditional destructive techniques used to characterize their properties as a function of manufacturing parameters are expensive and time-consuming. A novel non-destructive, data-driven method leveraging ultrasonics and machine learning techniques to tailor the properties of thermosets as a function of the manufacturing parameters is demonstrated. Thermoset epoxy samples with varying curing temperatures (15–40 °C) and curing agent amounts (±40%) were manufactured and tested. Their curing kinetics were monitored by determining the sound speed in the material in real time, while the longitudinal modulus of the samples was determined post-cure. Machine learning models were developed using a k-nearest neighbors algorithm. These models were implemented to predict the curing and final elastic properties using the manufacturing parameters, i.e., stoichiometry and curing temperature, and vice versa. Understanding and modeling how these parameters affect the cure kinetics and final properties will allow for efficient and reliable optimization of thermoset tailoring and manufacturing.

## 1. Introduction

Thermoset epoxy resins are heavily utilized constituents in composites, coatings, and adhesives due to their desirable mechanical, electrical, and thermal properties [1,2,3,4,5]. They are commonly adapted for use in specific applications by tailoring their properties to withstand the intended operating environment, which is achieved by varying manufacturing parameters such as stoichiometry, curing temperature, and fillers. For example, altering the stoichiometry of a resin by varying the amount of curing agent can significantly affect its mechanical properties; Excess curing agent can decrease the fatigue crack propagation while increasing the fracture toughness [6,7]. However, changing the stoichiometry can also negatively affect the glass transition temperature or moisture ingression [8,9]. H. Wang et al. reported a decrease in glass transition temperature from 87.2 to 73.7 °C when decreasing the amount of curing agent by 20% in a bisphenol A epoxy resin with a diamine curing agent [10]. Changes in the molecular weight and crosslinking density have also been reported when the stoichiometry of the resin is varied [11,12].

Polymer chemistry plays a crucial role in how the crosslinking of the resin behaves during the curing process. When the epoxy and hardener are mixed, curing begins with the formation of microgel particles that contain low molecular weight. These chains then link together, forming a continuous phase. As this process continues, vitrification occurs, causing a decline in the reaction rate, making further crosslinking reliant on diffusion and mobility [3,13]. However, some uncrosslinked molecules cannot react, which can prevent the curing process from being fully completed. This causes the mechanical, thermal, or electrical properties to be negatively affected. This limitation typically arises unless the epoxy is heated to its glass transition temperature since curing the epoxy at a higher temperature increases the mobility of the chains and molecules in the material. [14]. To reduce the likelihood of unreacted amine, one effective strategy is to use an excess of epoxy during the mixing process [11]. However, M. A. Andres et al. showed that while epoxy-rich samples have lower strength and increased brittleness, amine-rich samples result in polymers with increased flexural strength and ductility [15]. Therefore, it is essential to have a comprehensive understanding of how the elastic properties and curing process of resins behave as a function of stoichiometry and curing temperature to effectively tailor resin properties.

Mechanical properties and curing kinetics of resins are commonly characterized via destructive testing techniques such as dynamic mechanical analysis (DMA) [16,17], differential scanning calorimetry (DSC) [18,19], and tensile testing [20,21]. However, these techniques are difficult to implement correctly due to strict sample preparation requirements. They also utilize costly, lab-type equipment. In addition, because the samples are destroyed while evaluating their properties, it is not possible to characterize mechanical properties or curing kinetics in real-time. In contrast, ultrasonic non-destructive evaluation (UNDE) determines mechanical properties and curing kinetics via variations in sound speed through the polymer in real-time during the curing process.

UNDE uses two piezoelectric ultrasound transducers fixed to opposing outer walls of the curing container: one functions as a transmitter, which emits an acoustic burst and the other as a receiver that measures the burst after it has traveled through the sample. During transitional stages of polymerization, the sample’s matrix undergoes progressive stiffening, which alters the mechanical properties, increasing the sound speed [22]. Consequently, measuring changes to the sound speed during curing enables evaluation of the mechanical properties in real time. Additionally, because measurements can be performed in rapid succession and with high precision, UNDE is also used to evaluate the phase transitions during curing in real time [23]. Thus, cure kinetics are also captured. This technique has been used in previous work to characterize the influence of residual solvent in the curing mechanics and elastic properties of an epoxy adhesive [5], to determine the curing process of a thermoset resin with varying temperature and stoichiometry [24], and to determine the optimal stoichiometry in a polymer concrete composite [25]. Others have also used ultrasonic wave propagation to characterize polymers [26] and monitor the curing process of resins [27].

Although UNDE simplifies and extends the polymer characterization process, it is still often challenging, time-consuming, and costly to characterize the full range of possible properties a polymer can exhibit when cured. This would require manufacturing and testing a large number of polymer samples created from a high-dimensional set of manufacturing parameters. To overcome this challenge, in this work, we present a novel machine learning-based method to correlate the manufacturing parameters with the mechanical properties and the curing properties determined using non-destructive evaluation. Machine learning has been used to estimate and predict the properties of polymers [28,29]. For example, Pilania et al. implemented machine learning to accelerate the property prediction of polymeric materials using chemo-structural information and density functional theory [30]. I. Pavlovich et al. focused on using machine learning models to estimate and predict the physical characteristics of polymers using online available polymer databases [31], but these databases often do not have your composite of interest. This can become a problem for companies or research entities that use proprietary materials, which have never been manufactured and tested by other organizations. In the case of Z. Wan et al., they used a data set with 114 data points characterized via tensile fatigue testing to establish processing-property relationships [32]. However, manufacturing samples with specific dimensions and performing destructive testing is time- and material-consuming. This can be a challenge at the beginning stages of material development since there are often small material quantities available for testing. Our proposed method, combining machine learning with non-destructive testing, accelerates the characterization process, reduces non-recyclable material waste, allows for real-time testing, and avoids using expensive lab-type equipment.

More specifically, we train k-nearest neighbors regressors for four scenarios that may arise: (1) When the user wants to predict the longitudinal modulus and cure time resulting from a set of cure temperatures and stoichiometries; (2) when the user wants to predict which manufactured parameters are required for a set of desired longitudinal modulus and cure times; (3) when the user wants to predict the longitudinal modulus of a fully cured sample as the resin is in the early stages of cure; (4) when the user wants to predict the cure time of a resin based on the utilized manufacturing parameters and the longitudinal modulus. By accurately predicting how these parameters influence the resin’s mechanical performance and curing behavior, researchers and manufacturers can optimize formulations and processing conditions, leading to enhanced product performance and reliability. 

The proposed method consists of three distinguished stages, which will be expanded in Section 2:

1. Sample manufacturing: manufacture epoxy samples with varying curing agent-to-epoxy stoichiometric ratios and cure temperatures. This is further described in Section 2.1.
2. Sample characterization: evaluate the elastic moduli and curing process using ultrasonics as an NDE method. This is further described in Section 2.2 and Section 2.3.
3. Model training and testing: use a KNN regressor to build and test four different models for four different scenarios to correlate processing parameters with curing and elastic properties. This is further described in Section 2.4.

## 2. Materials and Methods

### 2.1. Materials and Sample Preparation

A thermoset, 2-part epoxy was utilized for this study. EPON 828 (Hexion), which is a liquid resin derived from difunctional bisphenol A/epichlorohydrin. This epoxy is commonly used for its good adhesive, chemical resistance, and mechanical properties [33]. An unmodified aliphatic amine, EPIKURE 3234 (Westlake Epoxy), was used as a liquid curing agent. For this resin/curing agent combination, a 13 phr (parts per 100 resin by weight) is recommended by the manufacturer. Samples with an amine-to-epoxy ratio, r, from 0.6 to 1.4 were manufactured, which corresponds to using ±40% curing agent. Six representative samples per ratio were cured at temperatures ranging from 15 to 40 °C in 5 °C increments. A total of 12 samples per cure temperature were tested, corresponding to 11 mix ratios with an additional r = 1 duplicate. A total of 72 samples, 12 per 6 cure temperatures, were evaluated and analyzed in this study. The resin and hardener were thoroughly mixed for 30 s and degassed under vacuum for 3 min. Each mixture was poured into a 12.5 × 12.5 × 45 mm^3^ polystyrene cuvette with a wall thickness of 1.25 mm. Samples were placed in an environmental chamber (SH-242, Espec) to be cured at each set temperature.

### 2.2. UNDE for Cure Kinetics

**Figure 1:**
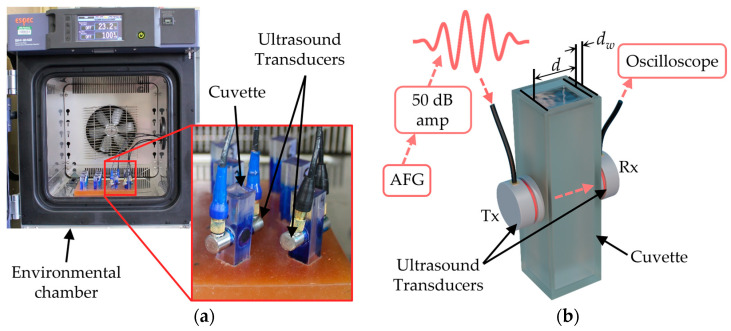
(**a**) Photograph of the UNDE testing system; (**b**) Schematic of the UNDE system with corresponding dimensional parameters and signal flow diagram.

UNDE was performed using two 2.25 MHz center frequency transducers (V106-RB, Olympus, Center Valley, PA, USA) aligned and adhered to the opposing outer walls of a cuvette as seen in Figure 1a. An excitation signal, 2.25 MHz, 5-cycle sine burst at 0.5 volts peak-to-peak, is transmitted by a function generator (AFG31052, Tektronix, Beaverton, OR, USA) to the emitting transducer. The signal is tapered with a Tukey window (cosine fraction α = 0.4) to minimize uncontrolled spectral leakage [34]. To promote signal transmittance through the cuvette walls and to differentiate signal-to-noise components within the measurement, signal amplification of 50 dB RF is attained using a power amplifier (2100 L, Electronics & Innovation, Rochester, NY, USA). The burst is then measured at the opposing transducer using an oscilloscope (MDO32, Tektronix). The emitted burst and measured signal are time-synced and saved via LabVIEW every 30 s for 24 h. A periodic measurement of the longitudinal travel time of an acoustic burst through the sample provides a quantifiable value that correlates to the kinetic stage of polymerization throughout the curing process.

To determine the longitudinal sound travel time *t* during the curing time, the method of cross-correlation between the excitation and the measured waveforms is used. This technique is described in detail in [34,35,36]. The longitudinal sound speed of the sample, *c*, is calculated as follows:

(1) $$c=\frac{d}{t-2\frac{d_{w}}{c_{w}}}$$

where *t* is the sound travel time between transducers, *d* is the thickness of the sample, *d_w_* is the thickness of the cuvette wall (1.25 mm), and *c_w_* is the longitudinal sound speed of the cuvette wall. This equation isolates the sound speed in the sample by removing acoustic travel time delays introduced by the cuvette walls. For this work, d = 10 mm, *d_w_* = 1.25 mm, verified via a micrometer, and *c_w_* = 2350 m/s, the sound speed in polystyrene [37].

**Figure 2:**
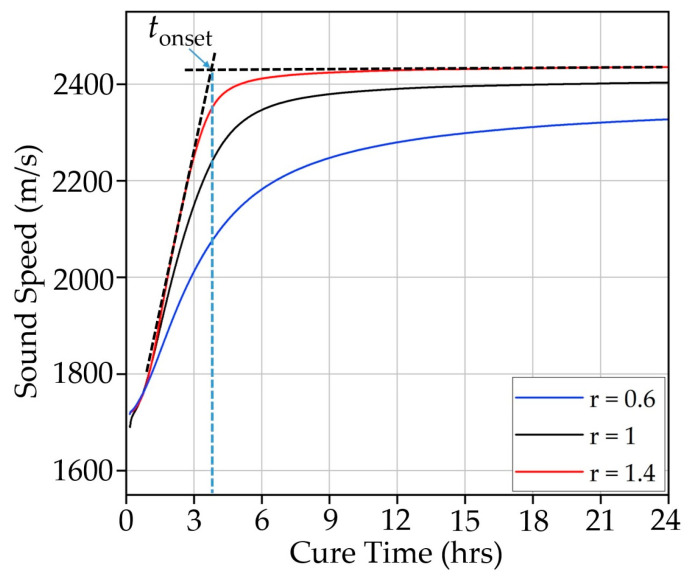
Sound speed during the first 24 h of cure of samples r = 0.6, 1, and 1.4 cured at 25 °C.

Figure 2 shows example sound speeds measured for samples with *r* = 0.6, 1.0, and 1.4 over a 24 h period at 25 °C. It is observed that as *r* increases, cure time decreases, and sound speed increases. In addition to gaining a qualitative understanding of cure behavior in real-time, using UNDE enables characterizing the phase transitions that the samples undergo during the curing process. For example, the time the samples take to enter the vitrification stage can also be obtained from the measured sound speeds. During this transition, the mobility of the polymer chains significantly decreases due to the formation of a three-dimensional crosslinked network that restricts the movement of the molecules. As the resin reaches the glass state, the reaction rate for crosslinking diminishes because the available mobility for the reactive groups is limited. Subsequent crosslinking becomes increasingly dependent on diffusion processes, which decreases the cure rate. This transition into the vitrification stage is characterized by the onset time *t_onset_*, which is determined by implementing the tangential method (see Supplementary Materials) as seen in the curve of the sample *r* = 1.4 in Figure 2.

### 2.3. Mechanical Properties

For this work, we use the longitudinal modulus *C_L_* to represent the mechanical property of a sample. *C_L_* is calculated as [23,27]

(2) $$C_{L}=\rho c^{2}$$

where ρ and *c* are the density and longitudinal sound speed of the material, respectively. The theoretical densities for each sample type were utilized, which were calculated using the densities of the resin and curing agent based on their ratios. Because the epoxy resin used is a dispersive medium, we use a combined cross-correlation frequency sweep technique after the samples are cured for 7 days to determine *c*. This technique is described in detail in [35,36,37]. A frequency sweep ranged from 1 to 2.3 MHz with a 0.1 MHz step.

### 2.4. Machine Learning Model

We use four parameters to train a k-nearest neighbors (KNN) regressor model for four scenarios (shown in Table 1) that may arise when attempting to cure a polymer with specific properties or limited resources. Thus, we train four KNN regressors using the following parameters:

- *T*: Temperature at which the resin was cured;
- *r*: Amine-to-epoxy stoichiometric ratio;
- *C_L_*: Longitudinal modulus obtained using Equation (2);
- *t_onset_*: Time at which the resin enters the vitrification stage.

Temperature (*T*) and stoichiometric ratio (*r*) are chosen as parameters since they are often changed to tailor the properties of polymers [3,9,10,14]. Then, the longitudinal modulus (*C_L_*) is chosen as a representative parameter of the elastic properties of polymers, while the time at which the resin enters the vitrification stage (*t_onset_*) is chosen as a representative parameter of the cure kinetics of the polymer.

For each scenario, the KNN regressor [38] is trained with a randomized 80/20 train/test split with the features as inputs and the target properties as outputs. Additionally, for each scenario, KNN models are trained with *k* = 1–10 neighbors and select the model for which *k* results in the smallest root mean square error (RMSE). This algorithm is selected for this proof-of-concept due to its simplicity and interpretability while also allowing adjusting the number of neighbors (k) for increased flexibility. KNN is also chosen due to its robustness against noise present in the data.

In scenario A, we use the manufacturing parameters *T* and *r* as features to estimate target features *C_L_* and *t_onset_*. This represents a situation where the user has limited control over the manufacturing parameters and seeks to know the resulting longitudinal moduli and the time it will take to reach those values. This enables understanding of the properties they are capable of obtaining within a limited range of temperature and ratio control.

In scenario B, we use *C_L_* and *t_onset_* as features to estimate the manufacturing parameters *T* and *r* required to achieve them. This represents a situation where the user requires specific mechanical properties and has limited time to obtain their cured product.

In scenario C, we use the manufacturing parameters *T*, *r*, and *t_onset_* as features to estimate the target feature *C_L_*. This represents a situation where all manufacturing parameters are known or restricted, and the user seeks to know the resulting mechanical property.

In scenario D, we use the manufacturing parameters *T* and *r* and the calculated *C_L_* as features to estimate the target feature *t_onset_*. This represents a situation where the user wants to predict when the resin will vitrify and can withstand a load, with a set of constrained manufacturing parameters.

## 3. Results and Discussion

### 3.1. Ultrasonics Testing

**Figure 3:**
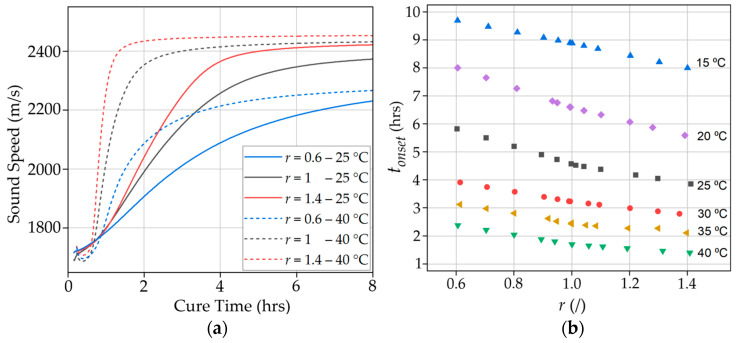
(**a**) Sound speed during the first 8 h of cure of the samples *r* = 0.6, 1, and 1.4 cured at 25 °C and 40 °C; (**b**) Onset times of all the tested samples.

Figure 3a shows the longitudinal sound speed measured in samples with *r* = 0.6, 1, and 1.4 at 25 and 40 °C over a 24 h period. We observe that the sample with the most curing agent (*r* = 1.4) cured faster and converged to a higher sound speed, which corresponds to a higher stiffness. This is because an excess amount of curing agent allows the present amines to find an epoxy to bond with, which accelerates the curing process and consumes the available epoxy. The opposite behavior is observed in the sample type having the least amount of curing agent (*r* = 0.6). In this case, part of the epoxy remains unreacted due to having a deficit of curing agent, which slows down the reaction. When comparing the curing temperatures, it can be observed that a higher temperature accelerates the curing process. This is due to increased mobility of the polymer chains and molecules at elevated temperatures [39]. In addition, the onset times correlate to when the samples have completed most of their crosslinking and have entered the vitrification stage. The onset times for the samples *r* = 0.6, 1, and 1.4 cured at 25 °C are 5.83, 4.52, and 3.86 h, respectively. In comparison, the onset times for the samples *r* = 0.6, 1, and 1.4 cured at 40 °C are 2.38, 1.70, and 1.41 h, respectively. Figure 3b shows the onset times of all the tested samples. Due to transducer availability, the longitudinal sound speeds of samples *r* = 0.6, 1, and 1.4 for each cure temperature were measured during curing and their onset times were determined as described in Section 2.2. For the remaining ratios, the onset times were approximated using linear interpolation based on the onset times of the samples *r* = 0.6, 1, and 1.4 at each temperature. For the samples tested, curing at higher temperatures accelerates the curing process and reduces the time required for the resin to reach the vitrification stage. It should be noted that the manufacturer recommends a cure time of seven days at room temperature (25 °C), but ultrasonics show that the samples have hardened and reached the vitrification stage during the first hours of cure, which varies with temperature. This information is useful to researchers and manufacturers that do in-field repairs during every season throughout the year and where the curing temperatures may vary based on the weather.

**Figure 4:**
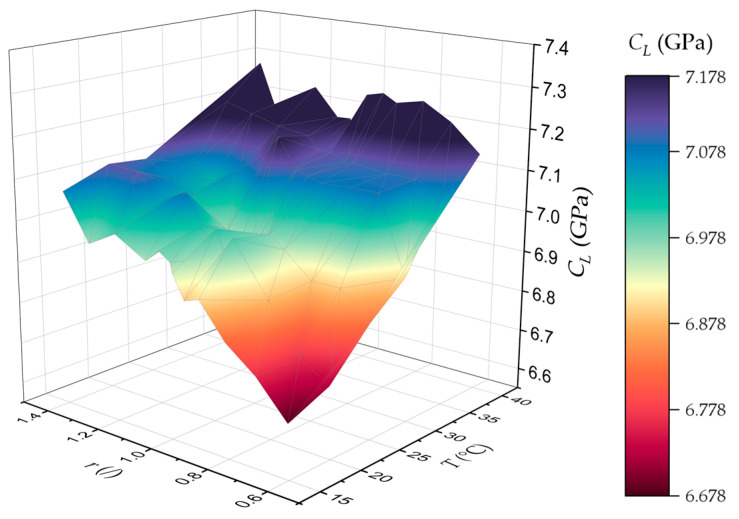
Three-dimensional plot of the longitudinal modulus of every tested sample as a function of the amine-to-epoxy ratio (r) and curing temperature.

Using Equation (2), the longitudinal modulus was calculated for every sample. Figure 4 contains a 3D plot of the longitudinal modulus as a function of the amine-to-epoxy ratio and curing temperature. Since the longitudinal modulus is dependent on the sound speed in the material, samples with higher amine-to-epoxy ratios had a higher longitudinal modulus due to the increased crosslinking of the available epoxy molecules. Similarly, higher curing temperatures increased the mobility of the molecules during curing for enhanced crosslinking, which caused the resin to have a higher longitudinal modulus.

### 3.2. Machine Learning

Table 2 summarizes the nearest neighbors (k) and RMSE obtained for each modeled scenario. The results for each scenario are described in the following sections from Section 3.2.1, Section 3.2.2, Section 3.2.3 and Section 3.2.4.

#### 3.2.1. Scenario A

**Figure 5:**
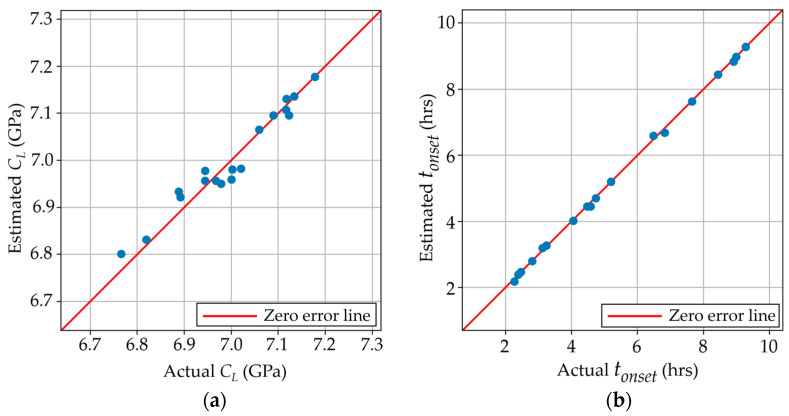
(**a**) Estimated vs. actual longitudinal moduli using the built model in scenario A; (**b**) Estimated vs. predicted onset times using the built model in scenario A.

Scenario A consisted of using the curing temperature and stoichiometric ratio as features to predict the longitudinal modulus and onset time during the curing process. Figure 5a shows the parity plot between the estimated and actual longitudinal moduli. The model predicted the *C_L_* across the whole range of values with an RMSE of 0.025 GPa. This model was also able to estimate the *t_onset_* with an RMSE of 0.063 hrs (~3.78 min), as observed in Figure 5b. Both models use k = 2 nearest neighbors. This model successfully predicted the longitudinal modulus and onset times with a known set of curing temperatures and stoichiometries with good accuracy. Predicting the mechanical property resulting from a set of manufacturing parameters is important to certify that a manufactured part can be safely used for a specific application. In addition, knowing the onset time, i.e., when the sample has reached the vitrification stage and can sustain the majority of the load that it is designed to sustain, can be beneficial in a manufacturing setting to reduce manufacturing or repair lead times.

#### 3.2.2. Scenario B

**Figure 6:**
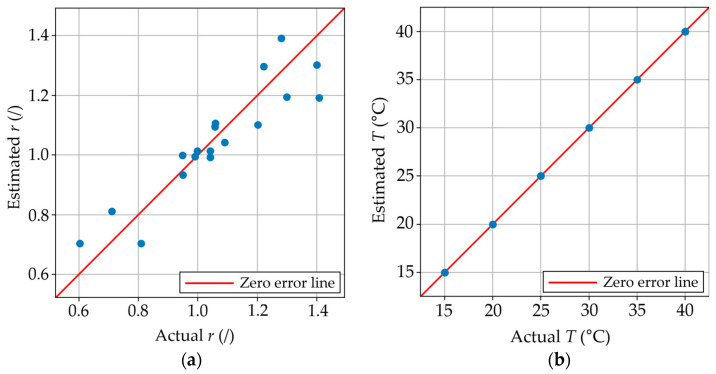
(**a**) Estimated vs. actual stoichiometric ratio using the built model in scenario B; (**b**) Estimated vs. actual curing temperatures using the built model in scenario B.

In the model in scenario B, the curing temperature and the stoichiometric ratio are predicted based on a desired set of mechanical and cure onset times. Figure 6a contains the parity plot between the estimated and actual stoichiometric ratios, where the model had an RMSE of 0.088. It can be observed in the parity plot in Figure 6a that despite having a small error, the variation between the actual and estimated values of *r* is lower in the region surrounding *r* = 1. The error in the *r* = 0.9–1.1 range is ~0.06, while the error is 0.115 in the upper and lower ranges. We hypothesize that this is caused by a larger number of samples existing in the region between *r* = 0.9 and 1.1 compared to the higher and lower ends of the range. The tolerances of these errors are highly dependent on the user and the application. For example, more samples can be manufactured and tested if a lower number is desired, while a lower number of samples can be used for the training to reduce the manufacturing/testing time and decrease material usage if a higher tolerance is acceptable. On the other hand, it can be observed in the parity plot in Figure 6b how the model accurately predicted the cure temperature with an RMSE of 0 °C. In addition, slight variations in the manufacturing procedure (i.e., mixing procedure by different individuals) can also introduce variability in the final product. The differences in error between the *r* and *T* predictions may be explained because 12 samples (one for each ratio) were manufactured and tested at each curing temperature, while only 5 samples per stoichiometric ratio (one for each curing temperature) were used to train and test the model. In both cases, a value of k = 1 resulted in the lowest RMSE. This prediction can be highly advantageous in determining the required manufacturing parameters to make a resin with a specific set of properties suited for particular manufacturing or repair scenarios.

#### 3.2.3. Scenario C

**Figure 7:**
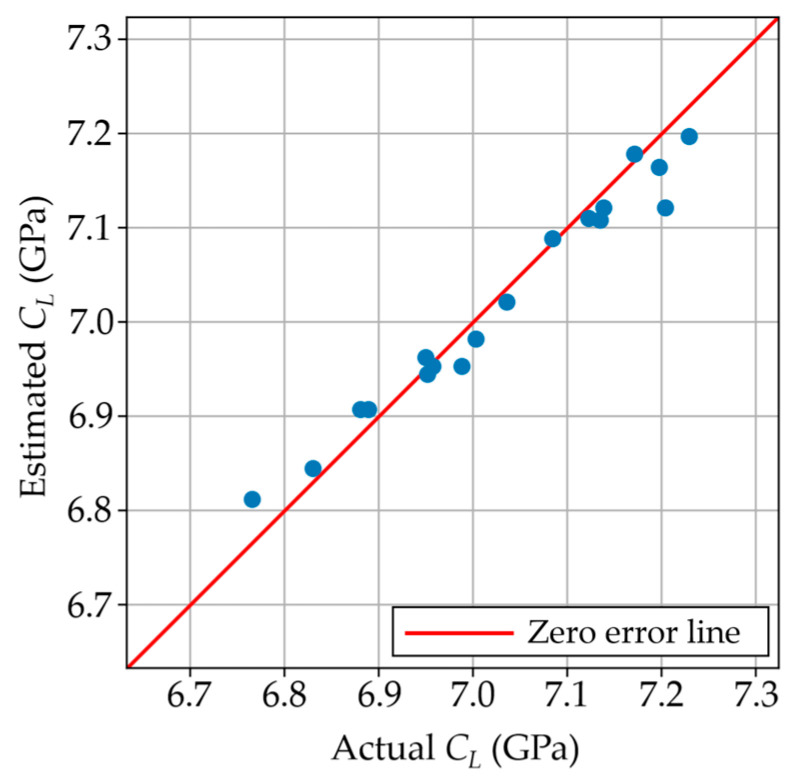
Estimated vs. actual longitudinal moduli using the built model in scenario C.

In this scenario, the longitudinal modulus of the cured samples was predicted utilizing the cure temperature, stoichiometric ratio, and onset time. Figure 7 shows the parity plot between the actual longitudinal modulus and the estimated values by the model. It can be observed that the model predicted the longitudinal modulus across the whole range of values with an RMSE of 0.029 GPa. In this case, a k = 4 resulted in the lowest RMSE. As mentioned, accurately predicting the longitudinal modulus in this scenario can be beneficial to avoid waiting until the resin is fully cured to predict its final elastic properties.

#### 3.2.4. Scenario D

**Figure 8:**
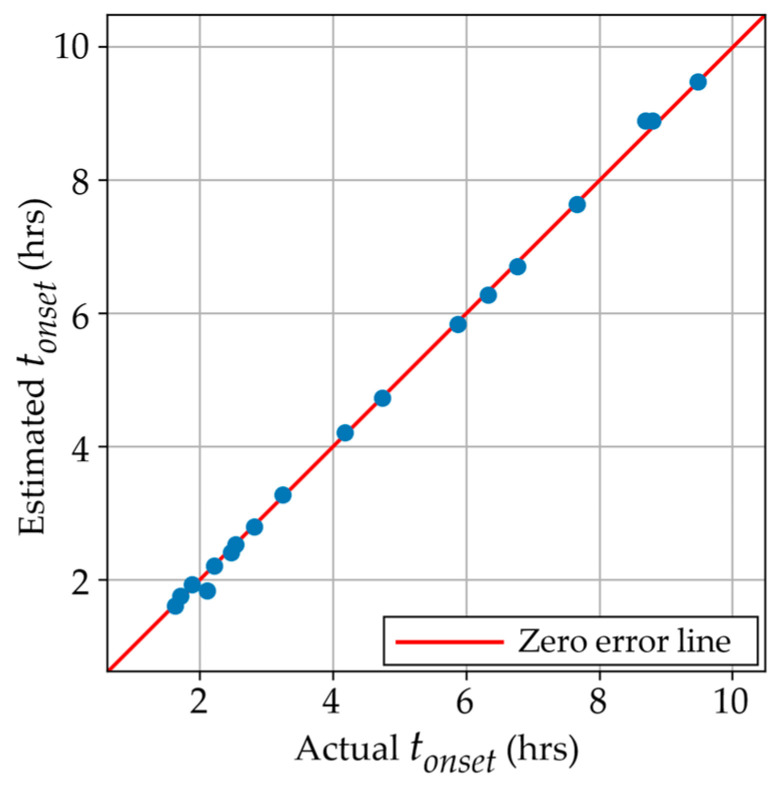
Estimated vs. actual onset times using the built model in Scenario D.

In scenario D, the onset time was predicted using the curing temperature, stoichiometric ratio, and the longitudinal modulus. As observed in the parity plot in Figure 8, this model successfully estimated the onset time across the available range with an RMSE of 0.089 h, which corresponds to an error of 5.5 min. As mentioned in Section 3.2.2, the error tolerances are highly dependent on the specific application in which the material will be implemented, and the number of samples manufactured and tested can be tailored by the user. In this case, a k = 2 resulted in the lowest RMSE. By accurately predicting when the material has hardened and can support the majority of its intended load, manufacturing times for structures can be significantly reduced. This allows components containing the resin to progress to the next stage in the manufacturing process knowing that they have reached the vitrification stage.

We note that there are a large number of possible material properties and manufacturing parameters that could be included in a trained model. It is also possible to train different ML regressors that may reduce errors. However, this paper is intended to provide a proof-of-concept for ML-based polymer curing predictions. Thus, other variables and ML algorithm optimization are left for future work.

## 4. Conclusions

This study presents a novel approach for tailoring the properties of thermoset polymers through the integration of ultrasonics and machine learning techniques. By systematically investigating the effects of curing temperature and stoichiometry on the curing kinetics and final elastic properties of epoxy resins, predictive KNN regressor models were developed for four epoxy resin manufacturing scenarios that may be encountered. Using non-destructive testing allows for high data throughput compared to traditional destructive testing, which is essential when training ML models. The UNDE results demonstrate that the curing process can be precisely monitored in real time, leading to significant insights into the relationship between manufacturing parameters and material performance without the need to destroy the sample. Low RMSE between the actual and estimated properties demonstrates that ML can be used to predict the properties and manufacturing parameters of thermoset polymers. The accurate predictions of longitudinal modulus and onset time not only enhance confidence in the performance of parts during the manufacturing process but also contribute to reducing lead times and enhancing efficiency in production and repair scenarios. The methods and findings of this research hold great promise for advancing the field of thermoset polymers, offering valuable tools for both researchers and manufacturers aiming to optimize materials for high-performance applications. Future work may explore the applicability of this approach to a broader range of thermoset systems, further enhancing the versatility and efficiency of polymer manufacturing processes.

## Figures and Tables

**Table 1:** Features and target properties used for each modeled scenario.

Scenario	Features		Target Properties
A	*T*, *r*		*C_L_*, *t_onset_*
B	*C_L_*, *t_onset_*		*T*, *r*
C	*T*, *r*, *t_onset_*		*C_L_*
D	*T*, *r*, *C_L_*		*t_onset_*
*T*: Cure temperature (°C)		*r*: Amine-to-epoxy ratio (/)	
*C_L_*: Longitudinal modulus (Pa)		*t_onset_*: Transition onset time (h)	

**Table 2:** Nearest Neighbors (k) and RMSE for each Scenario.

Scenario	Nearest Neighbors (k)	RMSE
A (*C_L_*)	2	0.025 GPa
A (*t_onset_*)	2	0.063 h
B (*r*)	1	0.088
B (*T*)	1	0 °C
C (*C_L_*)	4	0.029 GPa
D (*t_onset_*)	2	0.089 h

## Data Availability

The authors do not have permission to share data.

## Supplementary Materials

The following supporting information can be downloaded at https://www.mdpi.com/article/10.3390/polym17070895/s1.

## Abbreviations

The following abbreviations are used in this manuscript:

DMA	Dynamic Mechanical Analysis
DSC	Differential Scanning Calorimetry
UNDE	Ultrasonic Non-Destructive Evaluation
KNN	K-Nearest Neighbors
RMSE	Root Mean Square Error
ML	Machine Learning
