# Supplementary material for: Data-Driven Tailoring Optimization of Thermoset Polymers Using Ultrasonics and Machine Learning
Source: Polymers (Basel). 2025 Mar 27;17(7):895. doi: 10.3390/polym17070895 (PMC11991059; doi:10.3390/polym17070895)
Supplement: Supplementary file 1 [file polymers-17-00895-s001.zip › polymers-3527391-supplementary.pdf]

polymers

MDPI

Supplementary Materials

Tangent Method

Curve onsets are commonly determined using the Tangent method. In this scenario, t_{onset} is computed as the time at which the maximum rate of change tangent line intersects the final cure tangent line (See Figure 2). This is often accomplished graphically by manually placing estimated tangent lines and approximating the intersect, which can be error-prone. To minimize potential error due to tangent estimation and to use a consistent method, in this work, instead, the intersect time formula is derived and used to compute t_{onset} directly. The derivation and formula are provided here. Let

$$y_1(t) = \left.\frac{dc}{dt}\right|_{t_m} t + b_m, \tag{S1}$$

$$y_2(t) = \left.\frac{dc}{dt}\right|_{t_f} t + b_f \tag{S2}$$

represent the maximum rate of change and final cure tangent lines, respectively. Here, the notation $\cdot|_{t_0}$ evaluates the derivative of the sound speed c at a time $t = t_0$. t_m and t_f are the time at which the maximum rate of change occurs and the final cure has been completed, respectively. b_m and b_f are the tangent line intercepts, which are computed as

$$b_m = c(t_m) - \left.\frac{dc}{dt}\right|_{t_m} t_m \tag{S3}$$

$$b_f = c(t_f) - \left.\frac{dc}{dt}\right|_{t_f} t_f. \tag{S4}$$

To calculate t_{onset} we set $y_1(t) = y_2(t)$ and solve for t, which yields

$$t_{onset} = \frac{\left(c(t_f) - c(t_m)\right) - \left(\left.\frac{dc}{dt}\right|_{t_f} t_f - \left.\frac{dc}{dt}\right|_{t_m} t_m\right)}{\left.\frac{dc}{dt}\right|_{t_m} - \left.\frac{dc}{dt}\right|_{t_f}}. \tag{S5}$$

However, it is often the case after curing has finished that $\left.\frac{dc}{dt}\right|_{t_f} = 0$. Thus, Equation (S5) reduces to

$$t_{onset} = \frac{c(t_f) - c(t_m)}{\left.\frac{dc}{dt}\right|_{t_m}} + t_m. \tag{S6}$$

The variables used in this equation are relevant to the work presented in this manuscript. However, they can be substituted by any variable that corresponds to the curves obtained in other applications.
